# Acute Intoxications Involving α-Pyrrolidinobutiophenone (α-PBP): Results from the Swedish STRIDA Project

**DOI:** 10.1007/s13181-018-0668-2

**Published:** 2018-06-19

**Authors:** Lisa Franzén, Matilda Bäckberg, Olof Beck, Anders Helander

**Affiliations:** 1 Swedish Poisons Information Centre, SE-171 76 Stockholm, Sweden; 2 Department of Laboratory Medicine, Karolinska Institutet, Stockholm, Sweden; 3 Department of Clinical Pharmacology, Karolinska University Laboratory, Stockholm, Sweden

**Keywords:** α-PBP, New psychoactive substances, NPS, Cathinone, Intoxications

## Abstract

**Introduction:**

Many new psychoactive substances (NPS) introduced as recreational drugs have been associated with severe intoxication and death.

**Methods:**

Blood and/or urine samples were collected from intoxicated patients treated at Swedish hospitals that participated in the STRIDA project, a nationawide effort to address the growing problem of NPS. In patients undergoing evaluation for drug overdose, α-PBP was identified using liquid chromatography-mass spectrometry. Demographic and clinical data were collected during Poisons Information Centre consultations and retrieved from medical records.

**Results:**

From April 2013 to November 2015, 43 patients tested positive for α-PBP. However, α-PBP was never specifically mentioned during consultation but only confirmed analytically. The α-PBP concentrations ranged 2.0–13,200 ng/mL in urine and 2.0–440 ng/mL in serum. The patients were aged 19–57 (mean 34) years, 81% were men, and 73% were known drug addicts. All cases except 1 also involved other NPS and/or classical drugs. MDPV, α-PVP, and other pyrovalerone analogues were the most common other NPS (31 cases; 72%). CNS depressants were detected in 28 cases (65%), with benzodiazepines (16 cases) being most frequent. Main clinical characteristics were agitation/anxiety (59%), tachycardia (54%), and hypertension (37%), and 14 patients (33%) required monitoring in the intensive care unit of which 8 were graded as severe intoxications. No fatalities were reported.

**Conclusion:**

Patients with intoxication from α-PBP resembled those by NPS cathinones MDPV and α-PVP. As patients never specifically declared α-PBP intake and poly-drug intoxication was common, they may have been unaware of the actual substance taken.

## Introduction

The phenomenon of new psychoactive substances (NPS), where structurally modified variants of recreational drugs are introduced via online sale, has caused significant health problems during the last decade. Despite numerous reports of death and serious poisonings related to the use of NPS, recreational drug users continue to show interest in using these unclassified but poorly investigated psychoactive substances [1]. In 2014 and 2015, about 100 NPS were detected in Europe each year, and in 2016 the total number of drug substances monitored by the European Monitoring Centre for Drug and Drug Abuse (EMCDDA) exceeded 620. Although the number of NPS found in Europe has decreased over the past 2 years, the availability of NPS remains high [2].

Most NPS have belonged to the chemical classes synthetic cathinones and cannabinoids, but in recent years, the number of NPS opioids and benzodiazepines has increased [2, 3]. When NPS are placed under legal control, they are usually replaced with new structural variants. An example of this trend is pyrovalerone-related cathinones, which have caused high numbers of intoxications in Sweden and elsewhere. Popular NPS within this group have been 3,4-methylenedioxypyrovalerone (MDPV) and α-pyrrolidinovalerophenone (α-PVP) [4]. MDPV was first seized in Germany in 2007 [5] and it has since been involved in severe intoxications in several European countries [6, 7] and in the USA [8]. In contrast to most NPS, MDPV continued to appear on the Swedish drug scene several years after being banned, as shown by a high number of inquiries to the Swedish Poisons Information Centre (PC) and laboratory confirmed findings (Table 1) [9]. However, MDPV was gradually replaced by α-PVP, which has similar potency and effects and has caused severe intoxications and deaths [10, 11]. Since 2014, several other pyrovalerones have been introduced on the Swedish NPS market and involved in acute intoxications [12].

**Table 1:**
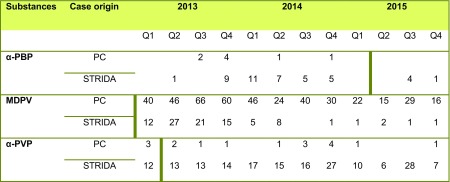
The yearly number (divided into quarters [Q]) of inquiries related to α-PBP, MDPV, and α-PVP exposures at the Swedish Poisons Information Centre (PC) and the numbers of analytically confirmed acute intoxication cases from the Swedish STRIDA project. The time that substance regulation entered into force in Sweden is indicated by a bold vertical line (MDPV was regulated already in 2010)

**Fig. 1:**
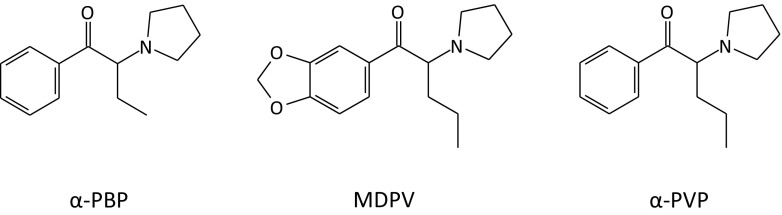
Chemical structures of α-PBP, MDPV, and α-PVP

α-Pyrrolidinobutiophenone (α-PBP) was one of the most prevalent pyrovalerone drugs that appeared among Swedish drug users after the legislation of MDPV and α-PVP (Fig. 1). α-PBP was first synthesized in the 1960s and included in a patent of α-pyrrolidinyl ketones and about 50 years later, it appeared as a recreational drug on the NPS market [13]. The first seizures of α-PBP reported to the EMCDDA originate from Finland in December 2011 and from Sweden in February 2013 [14]. On Internet drug chat forum, discussions regarding α-PBP started in March 2012 and lasted until January 2015, when the substance became legally controlled [15, 16].

In 2010, the Swedish Poisons Information Centre (PC) and the Karolinska University Laboratory in Stockholm started a nationwide project focusing on the NPS problem in Sweden (the Swedish project acronym is “STRIDA”). The overall aim of the STRIDA project was to assess the occurrence and trends of NPS use and collect clinical information about associated symptoms, toxicity, and health risks. Since the start of the project, many different NPS have been identified and toxicity data reported together with analytical data [17, 18]. This report presents clinical and bioanalytical data from drug intoxications involving α-PBP enrolled in the STRIDA project from April 2013 until November 2015.

## Methods

### Patient Inclusion Criteria and Samples

The PC receives telephone inquiries regarding acute intoxications from hospital caregivers and the public nationwide. When clinicians consulted the PC regarding intoxicated patients with admitted or suspected intake of NPS presenting in emergency departments (ED) and intensive care units (ICU), they were invited to participate in the STRIDA project including a free-of-charge drug analysis. If accepting to take part, blood and urine samples were collected for toxicological investigation; venous blood was used for the preparation of serum and urine was collected in tubes without additives according to established drug testing routines. The specimens were sent continuously to the Karolinska University Laboratory.

In each STRIDA case, information on the specific psychoactive substance and/or product brand name involved, the dose, time of intake, and route of administration were recorded during the consultation with clinicians. Copies of the medical record with full documentation of clinical signs and treatment were requested and sent to the PC retrospectively, and data on gender, age, symptoms, and treatment were retrieved. Drug addiction was established from documentation of ongoing use of illicit drugs, occasionally in combination with a positive hepatitis C diagnosis. The severity of intoxication was graded according to the Poisoning Severity Score (PSS) [19].

For the scientific evaluation, all clinical and laboratory data were anonymized and linked to the individual case only by a code number. For the present study, all patients positive for α-PBP in serum and/or urine from April 2013 until November 2015 were included, regardless of whether they also involved additional NPS or classical drugs of abuse.

The STRIDA project is conducted in accordance with the Helsinki Declaration, and it has been approved by the regional ethical review board (Nr. 2013/116–31/2).

### Laboratory Analysis

On arrival to the laboratory, serum and urine specimens were kept refrigerated until subjected for analysis which was typically done on the same or following day. Storage of specimens over longer time was done at − 80 °C. Measurement of psychoactive substances was carried out using liquid chromatography-mass spectrometry (LC-MS) or LC-high-resolution MS (LC-HRMS/MS) multi-component methods, as detailed elsewhere [20, 21], based on recording signals for two product ions for each analyte in positive selected reaction monitoring (SRM) mode. The LC-HRMS method is regularly updated with new substances, as they emerge on the recreational drugs market and reference material becomes available.

For analysis of urine, a 100-μL aliquot was diluted fivefold with 400 μL internal standard solution (methamphetamine–d5 in 0.1% formic acid). Serum samples were prepared by mixing 100 μL serum with 50 μL internal standard (pethidine-d5) solution, followed by addition of 400 μL acetonitrile. After mixing and centrifugation, 300 μL of the supernatant was transferred to autosampler vials and 2 μL injected into the LC system. Calibrators were prepared from blank samples. The measuring range covered 1.0 ng/mL to 1.0 μg/mL. Quantifications above the upper limit were done following dilution of samples with blank matrix. The exact mass of the monitored protonated molecular ion (M+H^+^) of α-PBP was 218.1539.

All urine samples were subjected to immunochemical screening for classical drugs of abuse, using CEDIA, DRI, and EIA reagents on an Olympus 640 or 680 Analyzer (Thermo Fisher Diagnostics), as detailed elsewhere [17, 18].

## Results

### Poisons Information Centre Inquiries—Reported Exposures

During the 20-month study period from April 2013 to November 2015, the PC received a total of 2931 consultations regarding suspected NPS intoxications of which 459 concerned pyrovalerone analogues. MDPV was by far the most common pyrovalerone mentioned in the PC query statistics (77%), but except for eight cases, the inquiries never related specifically to α-PBP exposure. Six of the cases suspected to involve α-PBP were inquiries from caregivers, but patient samples for the STRIDA project were never collected and α-PBP exposure could therefore not be confirmed (Table 1).

### The STRIDA Project—Analytically Confirmed Drug Exposures

The first α-PBP-positive patient identified in the STRIDA project appeared in April 2013. From then until November 2015, 43 ED/ICU patients tested positive for α-PBP of which 28 (65%) were from 2014. Intake of α-PBP was analytically confirmed in urine samples (*n* = 37) or, when urine was missing, in serum (*n* = 6). The α-PBP concentration ranged from 2.0 to 13,200 ng/mL in urine and from 2.0 to 440 ng/mL in serum. Sampling of blood (serum) and urine had been performed within 2 h after hospital arrival in 33 cases, within 5 h in another 3 cases, and in 2 cases after 16 and 36 h, respectively (information on sampling time was missing in 5 cases).

In 35% of the α-PBP-positive cases, MDPV was the drug reported by the patient or suspected by medical staff, and in almost half of those, intake of MDPV was confirmed. α-PBP was the only psychoactive substance detected in 1 case (2%). In the remaining 42 cases (98%), α-PBP was detected together with other NPS and/or classical CNS stimulants and depressants. The pyrovalerone analogues MDPV and α-PVP were the most common additional substances detected (31 cases, 72%) with α-PVP being the single most common (25 cases). Classical CNS stimulants were detected in 12 cases (28%) and other NPS in 14 (33%). CNS depressants were detected in 28 cases (65%) with benzodiazepines (16 cases) being most frequent, followed by ethanol (15 cases). In 58% of the cases, more than 3 additional substances were detected along with α-PBP. In 5 of those, 8 substances besides α-PBP were detected. A complete list of the analytically confirmed additional psychoactive substances is presented in Table 2.

**Table 2:** Substances detected in the analytical investigation of serum or urine along with α-PBP (*n* = 43). Patients testing positive for substances related to treatment confirmed by medical records and consultation notes are excluded

Additional substances	*N*	% of cases
Alpha-pyrrolidinovalerophenone (α-PVP)	25	58
Ethanol (including ethyl glucuronide and ethyl sulfate)	16	37
Amphetamine	12	28
Tetrahydrocannabinoid (THC)	11	26
Methylenedioxypyrovalerone (MDPV)	9	21
Alpha-pyrrolidinopropiophenone (α-PPP)	7	16
Pregabalin	7	16
Diazepam/oxazepam	7	16
Buprenorphine	7	16
3-/4-MeO-PCP	6	14
Alprazolam (4-hydroxyalprazolam)	6	14
Pentylone	5	12
Clonazepam (7-amino-clonazepam)	4	9
Morphine/morphine alkaloids	4	9
Methadone	4	9
4-Fluoro-α-PVP	3	7
MDMA/MDA	3	7
Codeine	3	7
3-/4-Methylmethcathinone (3-MMC, 4-MMC)	2	5
Tramadol	2	5
Butylone	2	5
Bupropion	2	5
Methiopropamine	2	5
4-Methylethylcathinone (4-MEC)	2	5
Dextromethorphan	2	5
Flubromazepam	2	5
N-Ethylbuphedrone (NEB)	2	5
Butyrfentanyl	2	5
5-MeO-MiPT	2	5
5-MeO-NiPT	2	5
Lorazepam	2	5
Methylphenidate (including ritalinic acid)	2	5
Nitrazepam (7-aminonitrazepam)	2	5
Pentedrone	1	2
Midazolam (4-hydroxymidazolam)	1	2
Alpha-pyrrolidinohexiophenone (α-PHP)	1	2
Alpha-pyrrolidinopentiothiophenone (α-PVT)	1	2
4-Fluoroamphetamine (4-FA)	1	2
4-Fluoro-PV8	1	2
Methamphetamine	1	2
2-Fluoroamphetamine (2-FA)	1	2
PV8	1	2
2-Aminoethoxydiphenyl borate (2-APB)	1	2
3-/4-Fluoromethcathinone (3/4-FMC)	1	2
MT-45	1	2
4-Ethylmethcathinone (4-EMC)	1	2
Mephedrone	1	2
Meclonazepam	1	2
25C-NBOMe	1	2
Fentanyl	1	2
3C-P	1	2
Methoxphenidine	1	2
Pyrovalerone	1	2

### Demographic and Clinical Data

Medical records were retrieved in 41 of the 43 α-PBP-positive cases and used for evaluation of demographic and clinical data. The age range of patients was 19–57 (mean 34) years and 81% were men. In 31 cases (76%), the patients had a drug addiction known to the medical staff. Reported routes of drug administration were injection (21 cases, 51%), oral (5 cases), inhalation/smoking (3), nasal (2), or unknown (12 cases). Multiple routes of administration were reported by 2 patients. The amount of drug taken was specified in 7 cases and varied from 0.3 to 1 g. Of the 41 patients, 29 (70%) were transported to hospital by ambulance, and police assistance was needed in 9 cases. In the remaining cases, the patients came to the ED by self-referral (10%), bystanders (10%), or for other/unknown reasons (10%).

Main clinical characteristics on admission were agitation/anxiety, tachycardia (≥ 100/min), and hypertension (systolic blood pressure ≥ 140 mmHg), which were observed in 24 (59%), 22 (54%), and 15 (37%) cases, respectively. Other common signs and symptoms were dilated pupils (24%), a reduced level of consciousness (Reaction Level Scale (RLS) [22] > 2) (17%), hallucinations (12%), rhabdomyolysis (10%), and delirium (7%) (Table 3). The severity of intoxication varied from none (PSS 0) to severe (PSS 3), with 9 cases (22%) classified as minor (PSS 1), 20 (49%) as moderate (PSS 2), and 9 (22%) as severe (PSS 3) intoxications. Three patients (7%) were asymptomatic (PSS 0). No fatalities were reported.

**Table 3:** Clinical signs observed at any time during admission among 41 cases of acute intoxications involving α-PBP

Clinical features	*N*	% of cases
Agitation/anxiety	24	59
Tachycardia (≥ 100/min)	22	54
Hypertension (systolic blood pressure ≥ 140 mmHg)	15	37
Mydriasis	10	24
Reduced level of consciousness (RLS > 2)	7	17
Miosis	6	15
Hallucinations	5	12
Rhabdomyolysis (CK > 25 μkat/L [∼ 1500 U/L] or myoglobin > 3000 μg/L)	4	10
Diaphoresis	4	10
Delirium	3	7
Seizures	3	7
Hypokalemia (≤ 3.3 mmol/L or mEq/L)	3	7
Clinical significant hyperthermia (> 39.0 °C [102.2 °F])	2	5

*RLS* reaction level scale [22], *CK* creatine kinase

The duration of hospital stay was 1 day for 18 patients (44%; 8 were discharged within 4 h), 2 days for 16 (39%) patients, and ≥ 3 days for 5 (12%) patients. ICU monitoring was required in 9 (22%) cases of which 8 were graded as severe intoxications (PSS 3). Standard supportive treatment including diazepam was used in 20 cases where 5 patients also received haloperidol. In 7 patients, sedation with propofol was required of which 5 were intubated. All patients recovered.

## Discussion

Intoxications associated with MDPV and α-PVP have led to many hospital visits during 2010–2015 in Sweden, and information about such cases in the STRIDA project on NPS has previously been published [6, 11]. α-PBP is one of several pyrovalerone analogues that succeeded MDPV and α-PVP on the Swedish NPS market [12]. The results of the present study indicated that α-PBP appeared as an NPS for about 3 years, and disappeared after being classified as a narcotic drug in January 2015. That NPS are taken off the open online drug market in response to legislation has been a common scenario for many other substances [23, 24].

Pyrovalerone analogues are characterized by the cathinone structure with a pyrrolidine group with varying carbon length on the α-carbon (Fig. 1). They are potent and selective dopamine (DA) and norepinephrine (NE) reuptake inhibitors with no effect on monoamine release [25, 26]. By increasing the α-carbon chain length, the affinity and potency at the DA transporter of the α-pyrrolidinophenones increases, suggesting an increasing abuse potential [27]. α-PBP has been shown to block catecholamine transporters in a similar manner as MDPV and α-PVP, but with reduced potency [28]. The pyrovalerones have served as substitutes for amphetamines by drug addicts [29, 30], but if missing to consider the differences in potency and the need of dosing adjustments between different stimulants, the drug user may develop a severe toxidrome.

The clinical features noted in intoxications involving α-PBP resembled those reported for MDPV and α-PVP in the STRIDA project, with tachycardia, agitation/anxiety, and hypertension being the most prominent symptoms. The frequency of symptoms and the severity of poisoning were also similar, except that tachycardia and agitation seemed to be higher for α-PVP compared with α-PBP and MDPV [11]. For α-PVP, there were also 2 fatal cases. Publications on α-PBP intoxications are sparse but one case report with fatal outcome detected α-PBP (200 ng/mL) postmortem in cardiac blood along with MDPV (1200 ng/mL) [31].

The characteristics (i.e., gender, age, history of drug use) of patients positive for α-PBP were similar to previous findings in MDPV and α-PVP positive patients. The intoxicated patients in the present study were mainly men above 30 years of age with previous experience of drug use. Intravenous injection was the most common reported route of drug exposure. In most cases, α-PBP was detected along with other psychoactive substances, mainly other pyrovalerone analogues with α-PVP being the single most common. The high occurrence of poly-drug intoxications, especially involving MDPV and α-PVP, indicated that many of these patients were stimulant drug users. However, α-PBP seems to have been an anonymous player on the NPS market, as α-PBP was almost never the self-reported drug nor mentioned by clinicians during PC consultation. It has been indicated that pyrovalerone NPS have commonly been sold under the trade name “MDPV” [6, 11]. This may suggest that α-PBP was used as any stimulant substitute, and that the actual substance taken largely depends on availability and cost.

The high frequency of reported poly-drug use, in some cases to up to 8 additional substances besides α-PBP, also indicated that NPS users are not always aware of the specific substance they take, or that mixtures of drugs are distributed. Poly-drug use has been a common feature in previous NPS case series from the STRIDA project [18, 23]. This is consistent with an EMCDDA report on high-risk drug use (e.g., stimulant users who switch to NPS and users who inject NPS) that NPS mainly occur in a context of poly-drug use and that NPS are rarely reported to be the primary drug [32].

Our study has several limitations that need to be acknowledged. First, the method used to collect clinical data is not standardized among all participant hospitals which may result in an absence of reported clinical findings and treatment. Also, information on circumstances regarding drug exposure (amount of drug taken, route of administration) or the time passing between intake, admission, and blood/urine sampling may be missing or unreliable. As is common in other studies using data from a poison center database, some of the clinical information is unverifiable and not consistently recorded or missing. Finally, the high occurrence of poly-drug use in these cases limits our ability to associate the clinical signs and severity of poisoning specifically to α-PBP.

## Conclusions

The clinical features and patient characteristics in intoxications involving α-PBP resembled those observed for the structural analogues MDPV and α-PVP. Mixing α-PBP with other psychoactive substances was common and may have contributed to more severe intoxications. The fact that patients rarely declared α-PBP intake on admission, in combination with a high frequency of poly-drug intoxications, may suggest that they were mainly interested in a general stimulatory drug effect, rather than in α-PBP specifically.
